# Optimized Decellularization of a Porcine Fasciocutaneaous Flap

**DOI:** 10.3390/bioengineering11040321

**Published:** 2024-03-27

**Authors:** Elise Lupon, Aylin Acun, Corentin B. Taveau, Ruben Oganesyan, Hyshem H. Lancia, Alec R. Andrews, Mark A. Randolph, Curtis L. Cetrulo, Alexandre G. Lellouch, Basak E. Uygun

**Affiliations:** 1 Department of Plastic and Reconstructive Surgery, Institut Universitaire Locomoteur et du Sport, Pasteur 2 Hospital, University Côte d’Azur, 06300 Nice, France; elise.lupon@gmail.com; 2 Vascularized Composite Allotransplantation Laboratory, Center for Transplantation Sciences, Massachusetts General Hospital, Harvard Medical School, Boston, MA 02114, USA; corentin.taveau@gmail.com (C.B.T.); hlancia@mgh.harvard.edu (H.H.L.); arandrews11@live.com (A.R.A.); mrandolph@mgh.harvard.edu (M.A.R.); ccetrulo@mgh.harvard.edu (C.L.C.J.); alellouch@mgh.harvard.edu (A.G.L.); 3 Shriners Children’s Boston, Boston, MA 02114, USA; aacun@widener.edu (A.A.); roganesy@bidmc.harvard.edu (R.O.); 4 Center for Engineering in Medicine and Surgery, Massachusetts General Hospital, Harvard Medical School, Boston, MA 02114, USA; 5 Department of Biomedical Engineering, Widener University, Chester, PA 19013, USA; 6 University of Grenoble Alpes, CNRS, TIMC UMR 5525, EPSP, 38000 Grenoble, France; 7 Department of Plastic and Reconstructive Surgery, Massachusetts General Hospital, Boston, MA 02114, USA; 8 Innovative Therapies in Haemostasis, INSERM UMR-S 1140, University of Paris, 75015 Paris, France

**Keywords:** decellularization, recellularization, vascularized composite allotransplantation, soft tissue reconstruction, tissue engineering

## Abstract

Reconstructive techniques to repair severe tissue defects include the use of autologous fasciocutaneous flaps, which may be limited due to donor site availability or lead to complications such as donor site morbidity. A number of synthetic or natural dermal substitutes are in use clinically, but none have the architectural complexity needed to reconstruct deep tissue defects. The perfusion decellularization of fasciocutaneous flaps is an emerging technique that yields a scaffold with the necessary composition and vascular microarchitecture and serves as an alternative to autologous flaps. In this study, we show the perfusion decellularization of porcine fasciocutaneous flaps using sodium dodecyl sulfate (SDS) at three different concentrations, and identify that 0.2% SDS results in a decellularized flap that is efficiently cleared of its cellular material at 86%, has maintained its collagen and glycosaminoglycan content, and preserved its microvasculature architecture. We further demonstrate that the decellularized graft has the porous structure and growth factors that would facilitate repopulation with cells. Finally, we show the biocompatibility of the decellularized flap using human dermal fibroblasts, with cells migrating as deep as 150 µm into the tissue over a 7-day culture period. Overall, our results demonstrate the promise of decellularized porcine flaps as an interesting alternative for reconstructing complex soft tissue defects, circumventing the limitations of autologous skin flaps.

## 1. Introduction

Deformities created by birth defects, trauma, inflammation, and medical conditions including cancer, constitute a significant global health burden accounting for 11% of worldwide disability-adjusted life years [1] and can be corrected by reconstructive surgery. Standard reconstructive surgery techniques include autologous, pedicled, or vascularized tissue flaps to cover complex composite tissue defects with bone or tendon exposure and when no other local solution is possible [2]. Autologous flaps are removed from an uninjured site from the patient, which may lead to significant donor site morbidity (e.g., scar disunion, loss of muscle function, scarring, contracture, nerve injury, pain, etc.) [3,4,5]. This approach is limited by the availability of the donor sites, especially if the defect is large [2]. Some defects also require anatomical like-for-like replacement, which may cause difficulty to restore the complex three-dimensional anatomy with the autologous flaps [6,7,8]. To overcome these challenges, a number of skin substitutes and dermal matrices have been developed and commercialized, yet none have truly functioned as a true replacement of autologous flaps due to the lack of vascular bed in them [9,10,11].

One approach to address the challenges of matrix-based reconstruction is to utilize acellular matrices with a pre-existing, microvascular network. Perfusion decellularization is an attractive technique to generate scaffolds that retain the vascular architecture and the extracellular matrix composition (ECM) of the native tissue [12]. In this technique, the tissue or organ is perfused through the vasculature with detergents, enzymes, and/or other chemicals to remove the cellular components, leaving an extracellular matrix scaffold that maintains the microvascular architecture of the native tissue [13]. Once cells are completely removed, the decellularized tissue can be repopulated with healthy cells to generate an engineered graft that is functional and transplantable [12,14]. Another advantage of engineering grafts using this technique is the possibility to use cells generated from the recipient tissue, such as those derived from patient-specific induced pluripotent stem cells [14], which would eliminate the need for immunosuppressive therapy frequently required for the patients.

The perfusion decellularization technique has been shown to be applicable to a number of different organs and tissues, including those used for reconstructive surgery [15,16]. A limited number of studies reported the decellularization of fasciocutaneous flaps from small [17,18,19,20] and large animals [21,22]. These studies have shown success in the removal of the cells using histological staining and/or the quantification of DNA content, and a few investigated the scaffold further for ECM characterization and biocompatibility [21]. Unfortunately, the confirmation of vascular patency and its subsequent effect on recellularization and in vitro and in vivo biocompatibility of the decellularized grafts has not yet been established. In this study, we tested three concentrations of decellularization detergent for effectively removing the cells in porcine fasciocutaneous flaps and characterized the decellularized tissues for ECM components, growth factors, vascular architecture, and their biocompatibility.

## 2. Materials and Methods

### 2.1. Animals

Adult female Yorkshire pigs (36–39 kg and 3.7–5.2 years old) were used in this study. The animals were housed at the Transplant Biology Research Center according to the Guide for the Care and Use of Laboratory Animals. The study was reviewed and approved by the Massachusetts General Hospital Institutional Animal Care and Use Committee.

### 2.2. Procurement of Fasciocutaneous Flaps

**Figure 1:**
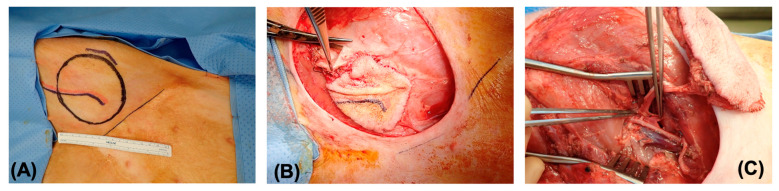
Procurement of the saphenous fasciocutaneous flap. (**A**) Preoperative marking of the flap; (**B**) dissection of the flap on its vascular pedicle; and (**C**) dissection extended to the femoral pedicle.

All specimens were harvested from pigs under general anesthesia in the operating room of the Knight Surgery Research Laboratory located at the Massachusetts General Hospital. One flap was harvested from each side of the donor animal using a surgical procurement technique described previously [23] (Figure 1). Briefly, an intravenous injection of 100 IU/kg heparin was performed 5 min before ligation of the femoral vessels below the inguinal ligament with 2-0 silk suture. Once the free flap was removed, the femoral artery of the flap was cannulated with a 20 G angiocatheter and then flushed with 100 mL of heparinized serum. At the end of the procedure, the pig was euthanized in accordance with the American Veterinary Medical Association (AVMA) guidelines for the euthanasia of animals.

### 2.3. Preparation of Decellularized Fasciocutaneous Flaps

The freshly procured fasciocutaneous flap was placed in a perfusion chamber and its vascular pedicle was connected to a continuous pressure-controlled perfusion system. It consisted of a Masterflex^®^ L/S^®^ digital drive equipped with an Easy Load^®^ II pump head (Cole-Parmer, Vernon Hills, IL, USA), a bubble trap (Radnoti, Covina, CA, USA), and a reservoir for perfusate. The fluid flow was directed through pre-sterilized Masterflex^®^ L/S Platinum-Cured Silicone Tubing size 16 (Cole Parmer, Vernon Hills, IL, USA). A pressure transducer was connected to the inlet tubing in shunt with the arterial catheter (BD Angiocath 20 G, Franklin Lakes, NJ, USA) to monitor the vascular pressure which was maintained at 90 mmHg throughout the perfusion by adjusting the flowrate.

**Figure 2:**
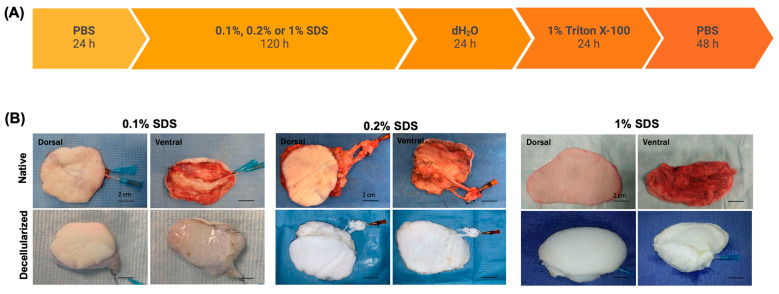
Decellularization of fasciocutaneous flaps. (**A**) Schematic of the perfusion decellularization protocol. Solutions and perfusion duration are indicated. The flow was maintained around 80–90 mmHg throughout the protocol. (**B**) Images of the flaps before (native) and after (decellularized) decellularization from dorsal and ventral views.

The decellularization was achieved by perfusion of the flaps with a series of solutions through a duration of 10 days (Figure 2). First, the flaps were flushed with phosphate buffered saline (PBS) for 24 h to remove any blood residue or other cellular debris. Next, they were perfused with sodium dodecyl sulfate (SDS) at different concentrations (0.1%, 0.2%, or 1% *w/v* in water, 3 flaps for each concentration) to begin decellularization for 120 h. At 48 h, de-epithelialization was performed manually using forceps. Perfusion with SDS was followed by a wash with distilled water (dH_2_O) for 24 h and 1% Triton X-100 for 24 h to remove any residual cellular debris and SDS (Supplemental Figure S1). Finally, the flaps were rinsed with PBS for 48 h and kept in sterile PBS at 4 °C for further analysis.

### 2.4. Measurement of DNA, Collagen, Glycosaminoglycans and Growth Factors in Decellularized Flaps

The center and the edge of the decellularized and native flaps were biopsied using a 3 mm biopsy punch both on the epidermal and subcutaneous sides and analyzed for DNA, collagen, and glycosaminoglycan (GAGs) content.

DNA was extracted from the tissues using DNeasy Blood & Tissue kit (Qiagen, Germantown, MD, USA), processing up to 25 mg of biopsies according to the manufacturer’s recommendations. Briefly, tissues were incubated overnight at 56 °C with proteinase K solution (40 mAU/mg protein). After adding buffer and ethanol, the mixture was transferred to a buffer-filled spin column, and repeated elutions were performed. Purified DNA from each sample was then quantified using the Nanodrop (Thermofisher Scientific, Waltham, MA, US) according to the manufacturer’s protocol. The final value was expressed as ng of DNA per mg wet tissue weight.

Similarly, total collagen content was measured using the total collagen kit (QuickZyme Biosciences, Leiden, The Netherlands); glycosaminoglycan (GAG) content was measured by a colorimetric assay following a protocol modified by Farndale et al. [24], as previously described [25]. The final values were expressed as micrograms per milligram wet tissue weight.

Growth factor analysis was performed using the RayBio^®^ Swine Growth Factor Antibody Array G-Series 1 kit (RayBiotech Life, Peachtree Corners, GA, USA) following the manufacturer’s recommendations (Supplemental Table S1).

### 2.5. Histological Analysis

The native and decellularized tissue samples were analyzed histologically using standard techniques. Briefly, the samples were fixed in 10% neutral buffered formalin for 24 h, processed, and then cut into sections at a thickness of 5 µm. The sections were stained with hematoxylin and eosin (H&E). Select samples were stained with Masson’s trichrome, according to standard protocols. The stained slides were scanned using Hamamatsu Nanozoomer Digital slide scanner (Hamamatsu Photonics K.K., Hamamatsu City, Japan).

### 2.6. Angiographic Imaging and Scanning Electron Microscopy

To visualize the vasculature in native and decellularized flaps, a contrast agent (Visipaque, GE Healthcare, Chicago, IL, USA) mixed with normal saline (1:2) was injected into the arterial pedicle using constant syringe pressure. Image acquisition was performed with a Powermobil C-Arm (Siemens, Munich, Germany). Images were exported in DICOM format and visualized with Osirix software 12.0 (Pixmeo, Bernex, Switzerland). This examination was performed on each flap before and after decellularization.

Scanning electron microscopy was performed for decellularized flaps at the Schepens Eye Institute core facility, supported by the NIH National Eye Institute Core Grant #P30EY003790. Briefly, the samples were dehydrated in graded ethanol solutions and dried at the critical point using a Samdri 795 critical point dryer (Tousimis, Rockville, MD, USA), then mounted on aluminum pedestals and chromed using a Gatan high-resolution ion beam coater (Gatan Inc., Pleasanton, CA, USA). Different surfaces of the samples were imaged using a JEOL JSM-7401F field emission scanning electron microscope (JEOL Inc., Peabody, MA, USA), allowing a qualitative assessment of the scaffold architecture.

### 2.7. In Vitro Biocompatibility of Decellularized Flaps

The decellularized flaps were tested for biocompatibility through in vitro cell culture. The decellularized samples of 0.5 cm^2^ were sterilized through incubation in sterile PBS supplemented with 4% ethanol and 0.1% peracetic acid for 24 h under agitation.

Sterile samples were placed in the wells of an ultra-low attachment 96-well plate for cell seeding. The flaps were preconditioned with serum-supplemented fibroblast base medium (ATCC, Manassas, VA, USA) in a cell culture incubator (37 °C, 5% CO_2_) for approximately 30 min before seeding. Primary human dermal fibroblasts (ATCC, Manassas, VA, USA) were cultured in a basic fibroblast medium (ATCC, Manassas, VA, USA) supplemented with a low-serum fibroblast growth kit (ATCC, Manassas, VA, USA). Cells were collected with 0.05% trypsin-EDTA (Thermofisher Scientific, Waltham, MA, USA) and resuspended in a culture medium for seeding onto the decellularized flaps. Cells were seeded at 1 × 10^5^ cells/well. They were cultured for 7 d at 37 °C and 5% CO_2_ and analyzed for proliferation using daily Presto Blue assay (Thermofisher Scientific, Waltham, MA, USA), calibrated to cell numbers according to the manufacturer’s instructions. At the end of 7 d culture, the scaffolds were fixed in 10% neutral buffered formalin and processed for histology. H&E-stained sections were analyzed using Image J (v. 1.53d) to determine the cell penetration depth.

### 2.8. Statistical Analysis

Graphical presentation and statistical analyses were performed on Prism 10 (GraphPad Software 10.1.1). For DNA, GAG, and collagen content, the means of all four biopsy locations ((1) epidermal, periphery, (2) subcutaneous, periphery, (3) epidermal, center, (4) subcutaneous, center) were analyzed separately using the two-way ANOVA test and Dunnett’s multiple comparisons to untreated flaps for statistical significance. The cell penetration and growth factor results were compared using Students’ *t*-test. The statistical significance was determined at a *p* value less than 0.5. Data are presented as mean ± standard error of the mean (SEM) for all analyses. The sample size was twelve or more for the biochemical and DNA assays and three for growth factor and cell penetration measurements.

## 3. Results

We procured and decellularized porcine saphenous fasciocutaneous flaps through a 10-day constant pressure perfusion with a series of solutions to enable the removal of the cells. The protocol consisted of several steps of washes with buffered solutions, deionized water, and detergents to facilitate the removal of cellular components and residual detergents in the final product. Decellularization was mainly achieved using sodium dodecyl sulfate, a strong anionic detergent, which was tested at three different concentrations, 0.1%, 0.2%, and 1% (*w/v*) (Figure 2A). The flaps were monitored for color change, an indication of cell removal, and any morphological changes throughout the decellularization. In all cases, de-epithelialization occurred within the first 24 h of SDS perfusion, which was sometimes facilitated by manual removal. The color of the flaps changed from pink to mostly white during decellularization. At the end of the perfusion, the flaps decellularized with 1% and 0.2% SDS turned completely white, whereas those treated with 0.1% SDS remained pink (Figure 2B).

### 3.1. The Effect of SDS Concentration on the Efficiency of Cell Removal

**Figure 3:**
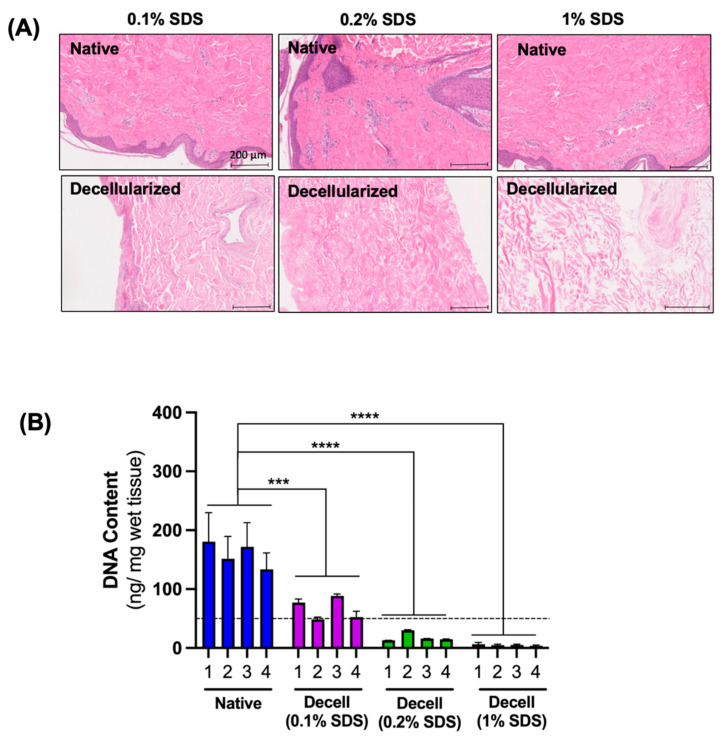
Removal of cells and DNA from the flaps as a result of decellularization. (**A**) Histological sections of native and decellularized flaps stained with H&E. (**B**) Quantification of DNA content in native and decellularized flaps. Biopsies were taken from four different locations: (1) epidermal, periphery, (2) subcutaneous, periphery, (3) epidermal, center, and (4) subcutaneous, center, and analyzed separately. Dashed line shows 50 ng/mg threshold which is an acceptable limit for complete decellularization. *** *p* < 0.001, **** *p* < 0.0001 by 2-way ANOVA and Dunnett’s multiple comparisons test of the means, *n* ≥ 12.

The efficiency of the cell removal was evaluated first by a visual assessment of the H&E-stained sections and second by measuring the DNA content of the decellularized flaps at the end of decellularization. For the DNA content measurement, the decellularized flaps were sampled at four different locations, on the epidermal and subcutaneous sides at both the center and the edge, to assess the uniformity of the cell removal. Microscopically, flaps decellularized using 0.2% and 1% SDS appeared completely eosinophilic with no residual nuclear stain, indicating the efficient removal of the cellular material. In the histological sections of the flaps decellularized using 0.1% SDS, there were no intact cells, but the nuclear stain remained diffusely present, indicating the inefficient removal of nuclear material from the flaps (Figure 3A). As a result of decellularization, the DNA content was significantly decreased, and the extent of removal increased with increases in the SDS concentration. On average, the DNA removal was 58.6 ± 3.6% with 0.1% SDS, 88.1 ± 5.5% with 0.2% SDS, and 96.9 ± 0.4% with 1% SDS. In all groups, the DNA removal was uniform with no statistically significant differences in DNA content among the four locations tested. In the flaps decellularized with 0.2% and 1% SDS, the final DNA content was below the threshold value of 50 ng/mg tissue, which is the commonly accepted value for complete decellularization (Figure 3B) [26].

### 3.2. Preservation of Extracellular Matrix Components and Vascular Microarchitecture

**Figure 4:**
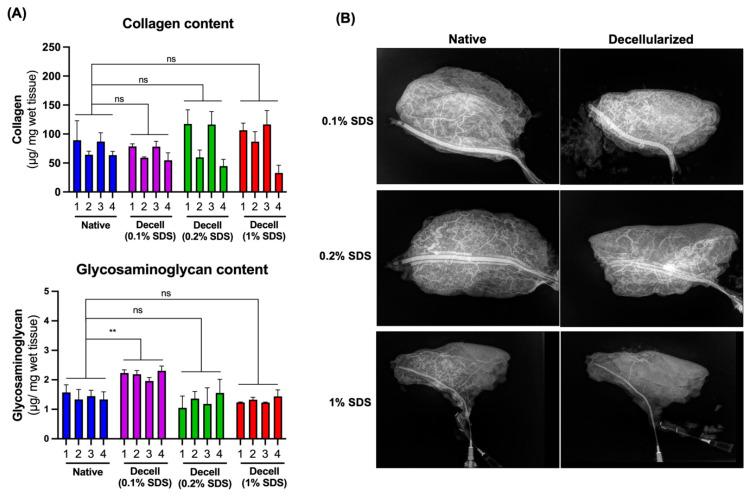
Preservation of extracellular matrix components and vascular microarchitecture in decellularized flaps. (**A**) Collagen (top) and glycosaminoglycan (bottom) content in native and decellularized flaps. Biopsies were taken from four different locations: (1) epidermal, periphery, (2) subcutaneous, periphery, (3) epidermal, center, and (4) subcutaneous, center, and analyzed separately. ns: not significant, ** *p* < 0.01 by 2-way ANOVA and Dunnett’s multiple comparisons test of the means, *n* ≥ 12. (**B**) X-ray imaging of the native and decellularized flaps with the contrast agent injected into the vasculature.

We analyzed the decellularized flaps biochemically to determine the extent of preservation of two extracellular matrix components, collagen and glycosaminoglycans. We found that collagen was maintained less uniformly in flaps decellularized with 0.2 and 1% SDS, with the subcutaneous layer containing significantly less collagen than the epidermal side. The differences were found to be statistically significant (*p* < 0.01). However, on average, the collagen content of the flaps in all decellularized groups remained the same as the native flaps; the differences were not statistically significant (Figure 4A). The average collagen content in the native flaps was 76.0 ± 14.0 µg/mg, 85.7 ± 37.3 µg/mg in the 1% SDS group, 84.6 ± 37.8 µg/mg in the 0.2% SDS group, and 67.7 ± 12.6 µg/mg in the 0.1% SDS group. The GAG content in the decellularized flaps was found to be preserved uniformly throughout the tissue with no statistical differences in the GAG content based on the biopsy location. The average GAG content in the flaps decellularized with 1% SDS was 1.33 ± 0.05 µg/mg tissue, 1.14 ± 0.11 µg/mg tissue with 0.2% SDS, and 2.20 ± 0.09 µg/mg tissue with 0.1% SDS. The average GAG content was 1.42 ± 0.62 µg/mg tissue in the native flaps, which was not statistically significant from those of the decellularized flaps except for the ones decellularized using 0.1% SDS (Figure 4A).

We visualized the vascular microarchitecture in the flaps before and after decellularization with 1%, 0.2%, and 0.1% SDS using angiography with contrast (Figure 4B). In the flaps decellularized with 0.1 and 0.2% SDS, the vascular bed remained relatively intact. However, decellularization with 1% SDS led to significant vascular damage and loss of vascular patency. In two of the three flaps tested at the highest concentration of SDS, the vascular perfusion was completely blocked.

### 3.3. Characterization of the Flaps Decellularized Using the Optimized Protocol

**Figure 5:**
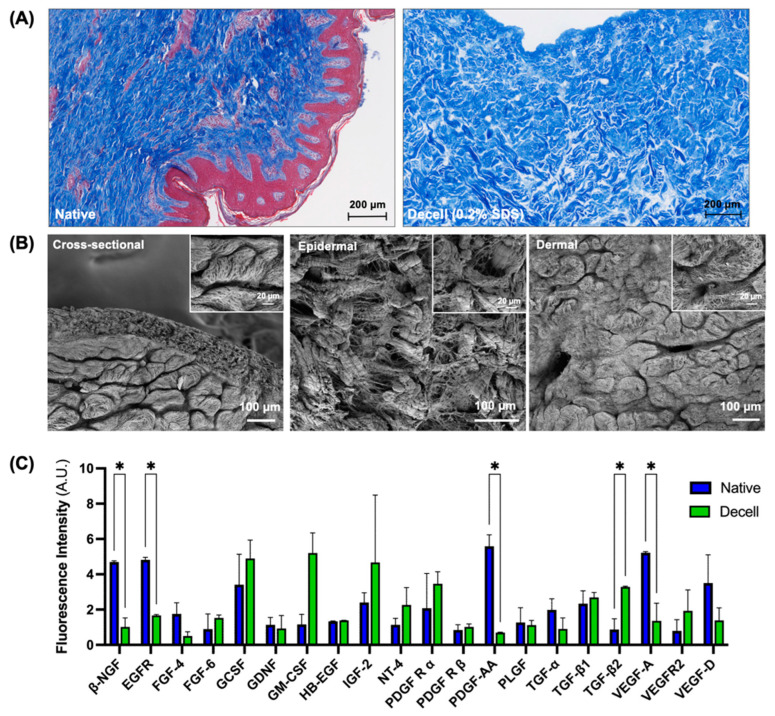
Characterization of the flaps decellularized using the optimized protocol. (**A**) Masson’s trichrome stain for collagen in native (left) and decellularized (right) flaps. (**B**) Scanning electron microscopy images of decellularized flaps; left to right: cross-sectional, epidermal, and dermal views. Insets show magnified views of these sections. (**C**) Retention of growth factor content in decellularized flaps in comparison to native flaps. Scale bars: (**A**) 200 µm, (**B**) 100 µm, and 20 µm (insets). * *p* < 0.05 and *n* = 3.

According to the analyses so far, decellularization using 0.2% SDS was found to be optimal, yielding decellularized flaps that retained the collagen and GAG in the ECM while preserving the microvascular architecture. We further characterized the flaps decellularized using 0.2% SDS. Masson’s trichrome stain showed an intact collagen structure with the complete removal of cells from the decellularized flaps (Figure 5A). An ultrastructural analysis using scanning electron microscopy also revealed the removal of cells throughout the decellularized flap. The extracellular matrix architecture was generally porous, displaying variations based on the location; the epidermal side had a more open structure while the dermal side had a tighter fibrous structure (Figure 5B). We also analyzed the decellularized flaps for growth factor content using a growth factor array and only plotted those that were detected in the native flaps (Figure 5C). Compared to the native flaps, there was no significant difference in the levels of the majority of growth factor levels following decellularization. However, there was significant decrease in the levels of β-nerve growth factor (β-NGF), epidermal growth factor receptor (EGFR), platelet derived growth factor subunit A (PDGF-AA), and vascular endothelial growth factor A (VEGF-A). Transforming growth factor β2 (TGF-β2) was the only growth factor found to be significantly increased in the decellularized flaps compared to the native flaps.

### 3.4. In Vitro Biocompatibility Assessment of Decellularized Flap

**Figure 6:**
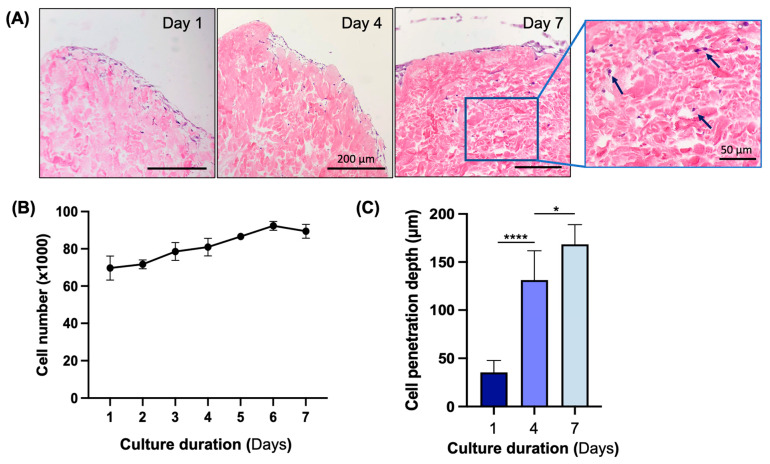
In vitro biocompatibility testing of decellularized flaps using fibroblasts. (**A**) H&E stain showing cell attachment and penetration through the dermal side of the scaffold on days 1, 4, and 7 after cell seeding. Arrows indicate cells found deep in the tissue. (**B**) Cell proliferation as measured with Presto Blue assay. (**C**) The quantification of cell penetration depth as distance from the epidermal surface of the scaffold over 7 days of culture. * *p* < 0.05, **** *p* < 0.0001 via Student’s *t*-test, *n* = 3. (Scale bars, main figure = 200 μm; scale bars, zoomed in image = 50 μm).

We tested the biocompatibility of the skin flaps decellularized using 0.2% SDS by culturing normal human dermal fibroblasts on 0.5 cm^2^ punch biopsies for 7 days. Over a culture period of 7 days, cells attached, proliferated, and penetrated deeper into the decellularized flaps. The H&E stain showed that cells formed a monolayer by day 1 of culture and continued to infiltrate into the decellularized flaps for the rest of the culture period (Figure 6A). The cell numbers as measured with the Presto Blue assay also increased steadily over 6 days and plateaued by day 7 (Figure 6B). The cell penetration depth increased significantly each day of measurement (Figure 6C), indicating the infiltration and engraftment of cells in the decellularized flaps.

## 4. Discussion

In this study, we report an optimized decellularization protocol for the preparation of porcine fasciocutaneous flaps for use in the engineering of vascularized composite allografts for reconstructive surgery. We found that constant pressure perfusion decellularization using 0.2% SDS resulted in tissues that maintained their microvascular architecture, which is a critical feature for the reconstruction of moderate to severe composite tissue defects.

There are several commercially available products that are derived from decellularized tissues (e.g., Alloderm^®^ LifeCell and ACell) and are FDA-approved to be used in plastic surgery to repair various tissues including the skin [10,27]. While these products are successful in providing some coverage of the defect, none have truly functioned as full-thickness skin comprising dermis and fascia. The reconstruction of severe defects requires the replacement of multiple layers of tissue with structural complexity and the need for transplantation with microsurgical anastomoses. The decellularization of fasciocutaneous flaps enables the development of a biological template that has the three-dimensional microarchitecture to recreate the complex tissue structure needed for reconstructive surgery. Indeed, our work demonstrated that the porcine fasciocutaneous flaps could be decellularized without damage to the overall structure of the tissue.

The optimized protocol that enabled the efficient decellularization of skin flaps utilized SDS as the main decellularizing detergent. SDS is a strong anionic surfactant that has been widely used in cell lysis and tissue and organ decellularization [28,29]. It is very efficient in removing the cells, but since it is a strong surfactant, it may also potentially remove critical extracellular matrix components from the decellularized tissue [14]. Triton X-100 was also used as the secondary detergent with the primary goal of removing the residual SDS from the tissue and was only tested at a single concentration [30,31]. We found that decellularization with 0.1% SDS perfusion was not effective in the removal of cells and DNA from the tissue when compared to those with 0.2% and 1% SDS. However, the histological assessment of the tissues after decellularization revealed that the ECM structure was much more open in the tissues decellularized with 1% SDS than the native tissue when compared to the other two concentrations of SDS, indicating some structural damage to the tissues. We did not find any statistically significant difference in collagen and GAG content as a function of the SDS concentration, indicating that exposure to 1% SDS did not remove any collagen and GAG content from the decellularized skin flaps. Structural damage was apparent when the vasculature in the decellularized skin flaps was imaged using X-ray angiography. While the extent of vascularization was the same between the native flaps and the decellularized flaps with 0.1% and 0.2% SDS, the presence of intact vasculature was very limited in the flaps decellularized with 1% SDS. As a result of these findings, we concluded that 0.2% SDS was ideal for the decellularization of porcine fasciocutaneous flaps.

There have been two other reports of porcine skin flap decellularization, both using SDS [21,22]. Using a very similar protocol, Jank et al. used 1% SDS to decellularize porcine groin fasciocutaneous flaps for 10 days [21]. The DNA removal efficiency was much lower than what we determined (77% vs. 88%) and they reported 56% retainment of GAG content. Xu et al. used 0.05% SDS to decellularize porcine radial forearm flaps for 5 days [22]. In their study, the DNA removal efficiency was comparable to our results at 86%, but the authors did not report the ECM component quantification and the biocompatibility of the grafts was not tested. While Jank et al. did not report the vascular patency of the decellularized flaps, Xu demonstrated venous outflow in the decellularized flaps, indicating the patency of the vasculature. However, the patency was not confirmed with any imaging technique; therefore, the uniformity of perfusion throughout the flap was unclear. Our results indicate that there were no significant differences in the DNA, collagen, and GAG content depending on the biopsy site, and that the contrast agent was uniformly distributed throughout the graft in X-ray angiography; these findings are indicative of vascular patency and uniform perfusion in the decellularized flaps.

The flaps decellularized with 0.2% SDS were further characterized for properties that are critical for subsequent recellularization studies. We first confirmed that the decellularized flaps had an open porous structure that enables the infiltration of cells during recellularization with SEM imaging. We also assessed the flaps for the presence of bound growth factors and confirmed that a majority of growth factors were maintained after the decellularization. Only b-NGF, EGFR, PDGF-AA, and VEGF-A were found to be significantly removed from the decellularized flaps. Each of these factors play a role in cell behavior, especially in recruitment and proliferation during wound healing [32,33]; therefore, it is important to consider supplementing these factors in the growth media during recellularization experiments, should it become necessary.

Finally, we tested the biocompatibility of the decellularized flaps by culturing dermal fibroblasts on small biopsy pieces. We found that fibroblasts attached, proliferated, and penetrated more than 150 µm deep into the tissues over a 7-day culture period. These results confirm that the decellularization process does not leave any cytotoxic residues that might negatively affect cell behavior. While the cell engraftment observed is encouraging, the extent of the repopulation we show here is not adequate to provide a functional graft. In order to improve the repopulation of the grafts, multiple different cell types including endothelial cells and keratinocytes should be used with different seeding strategies and cultured over extended periods of culture. For example, Jank et al. attempted to reconstruct the epidermis by culturing human keratinocytes on the decellularized flaps using air–liquid interface maturation and demonstrated barrier function using biotin [21]. They also report the endothelialization of the flaps by an infusion of human umbilical vein endothelial cells into the vascular pedicle using gravity perfusion with limited amount of success. We are currently working on the repopulation of the vasculature in decellularized flaps using perfusion-based seeding of the cells [34] to enable the complete coverage of both arterial and venous branches throughout the graft and prevent thrombosis. This will ultimately allow for long-term success upon transplantation.

## 5. Conclusions

We have successfully decellularized porcine fasciocutaneous skin flaps with an intact ECM and microvasculature using a low SDS concentration of 0.2%. Further characterizations of ECM-bound factors and in vitro biocompatibility after decellularization confirmed the utility of the prepared grafts for the generation of a full-thickness porcine fasciocutaneous flap of a clinically relevant size. These results are impactful since they represent the first steps towards creating engineered grafts for transplantation. Their clinically relevant size, preserved architecture, and ECM composition make them ideal for repopulation with healthy cells to recreate the native cellular architecture. Additionally, the repopulation of the grafts using differentiated cells from patient-derived stem cells will ultimately lead to engineered grafts that will eliminate the need for immunosuppression upon transplantation. Altogether, these porcine flaps represent a promising alternative for reconstructing complex soft tissue defects, circumventing the limitations of autologous skin flaps.

## Data Availability

Data can be provided by the corresponding authors on demand.

## Supplementary Materials

The following supporting information can be downloaded at: https://www.mdpi.com/article/10.3390/bioengineering11040321/s1, Figure S1: The chemical formulas and structures of detergents used for decellularization; Table S1: List of growth factors included in the growth factor array.
