# Supplementary material for: Optimized Decellularization of a Porcine Fasciocutaneaous Flap
Source: Bioengineering (Basel). 2024 Mar 27;11(4):321. doi: 10.3390/bioengineering11040321 (PMC11047669; doi:10.3390/bioengineering11040321)
Supplement: Supplementary file 1 [file bioengineering-11-00321-s001.zip › bioengineering-2919122-supplementary.pdf]

Supplemental Materials

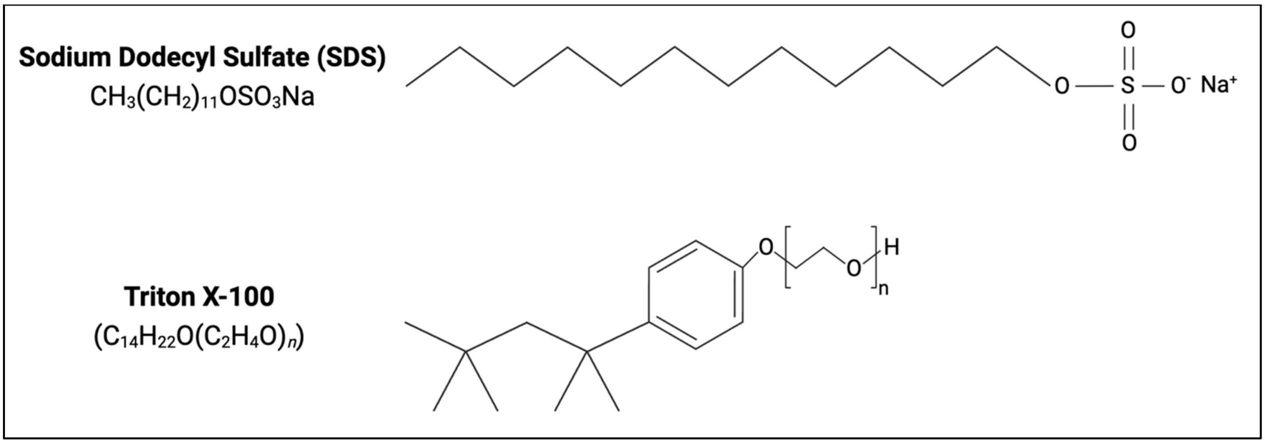

Figure S1 - The chemical formulas and structures of detergents used for decellularization.

Table S1 – List of growth factors included in the growth factor array.

**AREG**	Amphiregulin
**bFGF**	Fibroblast growth factor 2
**b-NGF**	Beta-nerve growth factor
**EGF**	Pro-epidermal growth factor
**EGF R**	Epidermal growth factor receptor
**FGF-4**	Fibroblast growth factor 4
**FGF-6**	Fibroblast growth factor 6
**FGF-7**	Fibroblast growth factor 7
**GCSF**	Granulocyte colony-stimulating factor
**GDNF**	Glial cell line-derived neurotrophic factor
**GM-CSF**	Granulocyte-macrophage colony-stimulating factor
**HB-EGF**	Proheparin-binding EGF-like growth factor
**HGF**	Hepatocyte growth factor
**IGFBP-1**	Insulin-like growth factor-binding protein 1
**IGFBP-2**	Insulin-like growth factor-binding protein 2
**IGFBP-3**	Insulin-like growth factor-binding protein 3
**IGFBP-4**	Insulin-like growth factor-binding protein 4
**IGFBP-6**	Insulin-like growth factor-binding protein 6
**IGF-I**	Insulin-like growth factor I
**IGF-I R**	Insulin-like growth factor 1 receptor
**IGF-II**	Insulin-like growth factor II
**M-CSF**	Macrophage colony-stimulating factor 1
**M-CSF R**	Macrophage colony-stimulating factor 1 receptor
**NT-3**	Neurotrophin-3
**NT-4**	Neurotrophin-4
**PDGF R a**	Platelet-derived growth factor receptor alpha
**PDGF R b**	Platelet-derived growth factor receptor beta
**PDGF-AA**	Platelet-derived growth factor subunit A
**PDGF-AB**	Platelet-derived growth factor subunit B
**PDGF-BB**	Platelet-derived growth factor subunit B
**PLGF**	Placenta growth factor
**SCF**	Kit ligand
**SCF R**	Mast/stem cell growth factor receptor Kit

**TGF-a**	Protransforming growth factor alpha
**TGF-b 1**	Transforming growth factor beta-1 proprotein
**TGF-b 2**	Transforming growth factor beta-2 proprotein
**TGF-b 3**	Transforming growth factor beta-3 proprotein
**VEGF**	Vascular endothelial growth factor A, long form
**VEGF R2**	Vascular endothelial growth factor receptor 2
**VEGF R3**	Vascular endothelial growth factor receptor 3
**VEGF-D**	Vascular endothelial growth factor D
